# Delimitating the Natural City with Points of Interests Based on Service Area and Maximum Entropy Method

**DOI:** 10.3390/e21050458

**Published:** 2019-05-02

**Authors:** Lingbo Liu, Binxin Xia, Hao Wu, Jie Zhao, Zhenghong Peng, Yang Yu

**Affiliations:** 1 Department of Urban Planning, School of Urban Design, Wuhan University, Wuhan 430072, China; 2 Department of Graphics and Digital Technology, School of Urban Design, Wuhan University, Wuhan 430072, China

**Keywords:** natural city, maximum entropy method, Reilly’s Law, uncertainty problems, head/tail breaks, points of interest

## Abstract

The natural city, which is essential to understand urban physical scale and identify urban sprawling in urban studies, represents the urban functional boundaries of the city defined by human activities rather than the administrative boundaries. Most studies tend to utilize physical environment data such as street networks and remote sensing data to delimitate the natural city, however, such data may not match the real distribution of human activity density in the new cities or even ghost cities in China. This paper suggests aggregating the natural city boundary from the service area polygons of points of interest based on Reilly’s Law of Retail Gravitation and the maximum entropy method, since most points of interests provide services for surrounding communities, reflecting the vitality in a bottom-up way. The results indicate that the natural city defined by points of interests shows a high resolution and accuracy, providing a method to define the natural city with POIs.

## 1. Introduction

The size of cites has multi-dimensional impacts on society, economy, and the environment, influencing energy and resource demands, urban congestion and biodiversity reduction [1,2]. The first step is to capture the boundaries of cities based on the density and distribution of human activities in a geographic context [3]. Although measuring human activities with traditional socioeconomic statistical data based on administrative boundaries is still prevalent, more and more research tends to identify urban boundaries with big data, such as remote sensing data, light imagery, road intersections, traffic connectivity, social network data, etc. [4,5,6]. Such kinds of urban boundaries refer to the concept of “natural city” inspired by Alexander. This widely influential urban theorist has indicated that the city is not a tree, but rather a dynamic, open half-network structure, which establishes a complex network perspective for understanding cities beyond their administrative boundaries [7]. The method for measuring natural cities admits the inherent diversity, non-linear dynamics and essential aesthetic characteristics of urban fractals underlying the complexity of human settlements [8,9,10,11], focusing on human activity data of everyday life [12]. 

**Figure 1:**
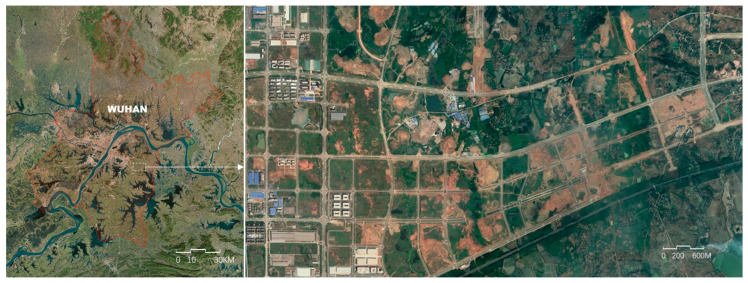
The pre-constructed street network in the east of Wuhan.

However, the ancillary data widely utilized to identify natural cities, such as street networks and remote sensing data, is mainly related to the physical environment, which may not match the real distribution of human activity density, for instance, in the new cities with pre-constructed street networks (Figure 1), or even the ghost cities in China [13,14]. The boundary identified by such physical data could be taken as a planned boundary, rather than a boundary of natural city.

As a bottom-up Volunteer Geographic Information source derived from daily life, points of interests (POIs) provide a large amount of spatial location information about facilities, such as stores, restaurants, pharmacies, and banks, capable of reflecting population density distributions [15] and understanding the size of the city [16]. Not only do commercial activities represented by POI density reflect the vitality of street network [17,18], but also the distance between POIs is highly related to the surrounding population [19]. 

Based on the heavy tail features of POIs in the urban complex system, Jiang has proposed a head/tail breaks method to extract urban boundaries from triangles in the Triangulated Irregular Network (TIN) generated by POIs, successfully measuring the natural city boundaries of European cities [20,21]. However, such a study gives rise to two main challenges: one is whether the TIN is suitable to construct the basic geographic unit for defining the natural city; the other is whether the breakpoint is reasonable since the head/tail breaks method takes the mean value as the threshold to aggregate triangles as the boundary. For instance, Rodriguez and Laio used 20% as the distance threshold in a FSDP decision graph [22], Wang introduced a minimum entropy method integrated with data field theory to seek the threshold value at the nadir for clustering [23], which could be taken as a derivation from the principle of maximum entropy.

**Figure 2:**
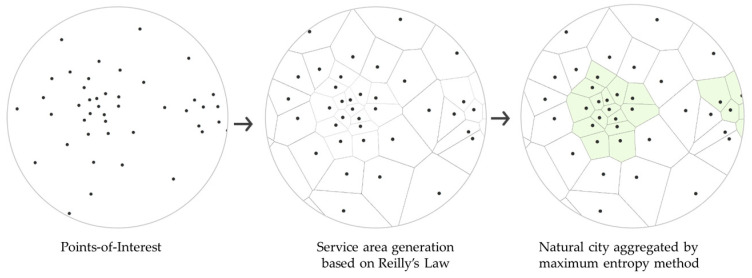
Workflow of SA-MaxEnt.

This paper proposes a SA-MaxEnt method that integrates Reilly’s Law of Retail Gravitation and the maximum entropy (MaxEnt) method to address these two problems (Figure 2). According to Reilly’s Law, customers tend to visit nearby retail centers, which attractiveness is determined by their size and distance to other retail centers, obeying the physical law of gravity [24], which indicates that the service area is tightly related to the surrounding population. Thus the service area that represents the attractiveness could be the better geographic unit to form the natural city boundary. Furthermore, the size of POIs could be ignored when the amount of certain kinds of POI is large enough, which turns the calculation of service area into a generation of a Voronoi diagram of POIs [25].

The maximum entropy method is then applied to compute the optimal threshold value for selecting Voronoi polygons to aggregate the nature city. As the population density varies from the city center to rural areas, the density of POIs would decline simultaneously along with the service area increasing. There might exist a reasonable breakpoint value of the distance to discriminate whether the POIs are located outside the urban area. The maximum entropy principle indicates that the probability distribution which best represents the current state of knowledge is the one with the largest entropy [26], thus this article assumes that the entropy value series for the ratio of all the polygons’ perimeters to all possible threshold values would achieve the maximum value at the optimal value. On the other hand, according to the 3σ principle of the Gaussian (normal) distribution presented by maximum entropy, the threshold range should be expanded to 3/$\sqrt{2}$σ [23].

Admittedly, such a hypothesis is not capable of delimitating a strict urban boundary based on the exponential decay of services, which would need further exploration with a fuzzy measurement method or ancillary data. The problem under discussion is within the scope of delimiting the natural city boundaries of a single city rather than cities in a national background which is performed in most studies, which focuses on the discussion of the extraction of minimum geographic units for city boundaries.

## 2. Methods and Materials

### 2.1. Service Area Generation Based on Reilly’s Law of Retail Gravitation 

There exist a large amount of service-based POIs such as retail stores, pharmacies, banks, and restaurants, which provide services locally for surrounding residents, varying in density in terms of nearby population. The spatial distribution of POIs may reflect the surrounding population density. Taking stores as an example, Reilly’s Law assumes that the attractiveness of two adjacent stores A and B is determined by their size (or service capacity) and the distance between them. The point where the two stores reach equilibrium becomes the breaking point to define their service distance (Equation (1)):

(1) $$L_{A}\;=\;\frac{L_{AB}}{1+\sqrt{S_{B}/S_{A}}}$$

**Figure 3:**
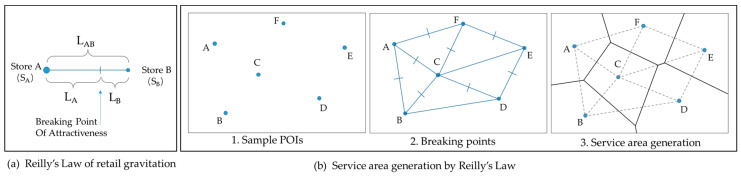
Service area generation based on Reilly’s law.

*L_A_* is the service distance of the store A in terms of the distance *L_AB_* between store *A* and *B*, *S_A_* and *S_B_* are the sizes of A and B, respectively (Figure 3a). While there exist numerous stores, the continuous connection of breakpoints between them constitutes the service area boundary.

Rather than POIs of a small amount like hospitals, which service area may be largely affected by its size or capacity, this paper took POIs in large amounts into account to neglect the influence of size, treating POIs as points of similar size. As for Equation (1), if *S_A_* = *S_B_*, then:

*L_A_* = *L_B_* = 1/2 *L_AB_* (2)

Thus the service area generation can be represented by the classic Thiessen polygon method (or Voronoi diagram): (1) each Thiessen polygon contains only one POI; (2) the distance between the points in the Thiessen Polygon and the central POI point is the closest compared to any other POIs; (3) the distance of the point on the edge of the Thiessen Polygon service area is equal to the central POI point on both sides (Figure 3b).

The generation of Thiessen polygons can be easily implemented by the Create-Thiessen-Polygons toolbox in ArcGIS. The service area polygons generated would be expected to show the fractal features as Jiang indicated [27], there will be a large number of small units in the urban area. Natural city boundaries can then be aggregated with polygons by an appropriate extraction method. 

### 2.2. Natural City Aggregated by Maximum Entropy Method

Entropy is a way of measuring uncertainty in the complex system. The concept of entropy in thermodynamics was first defined by Clausius, describing the energy unable to do work during the transferring procedure of energy [28]. Boltzmann used entropy as a measurement of disorder in thermodynamic statistics, which was introduced into information theory and then defined as uncertainty by Shannon [29]. Jaynes developed the principle of maximum entropy from Shannon’s expression [30]. Wilson introduced the principle of maximum entropy in urban and regional modeling [31]. The popular entropy weight method can also be taken as an application of the maximum entropy method [32,33,34].

As the maximum entropy method is suitable for solving uncertainty problems, this paper proposes to utilize it to seek the optimal threshold value for extracting service area polygons. Meanwhile, the perimeters of service area polygons were chosen as the factor to seek the threshold value, avoiding containing extremely narrow polygons with tiny areas while using area as the factor.

**Figure 4:**
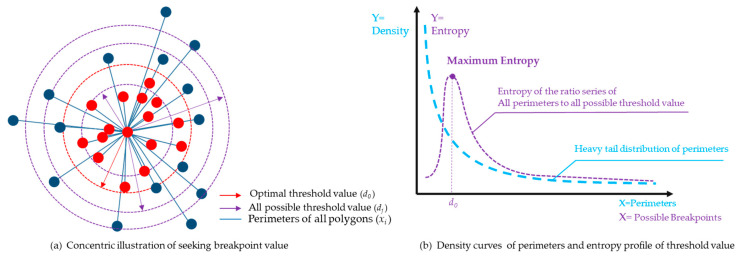
The assumption illustration for seeking threshold value by MaxEnt.

According to the principle of maximum entropy for uncertainty problems, the probability distribution which best represents the current state of knowledge is the one with the largest entropy. This paper assumes there may exist an optimal threshold value *d_0_* in random length data set D(*d_j_*) for all the perimeters data set X(*x_i_*), wherein the ratio function *F_d_ (x)* of *d_j_* and *x_i_* will achieve the maximum value at *d_0_* (Figure 4a). On the other side, the power law that perimeters of service area display in a form of heavy tail distribution can be transformed into Gaussian normal distribution by applying maximum entropy method (Figure 4b). Such transformation reflects the internal order, a self-organized feature of urban complexity system [35]. 

Because any perimeter *x_i_* that deviates from the threshold value *d_j_*, no matter smaller or larger than the threshold, will impose a more significant effect on the ratio of perimeters on a threshold value, thus the original ratio function *F_d_(x) = x_i_*/*d_j_* was revised as:

(3) $$F_{d}(x)=e^{\left|\ln x_{i}-\ln d_{j}\right|}$$

For each specific threshold value *d_j_*, the corresponding ratio sequences *P_ij_* of $F_{d_{j}}\left(x\right)$ for every perimeter *x_i_* is:

(4) $$P_{\mathrm{ij}}=\frac{F_{d_{j}}\left(x_{i}\right)}{\sum \;F_{d_{j}}\left(x_{i}\right)}$$

According to the definition of entropy, the total Shannon entropy *H_j_* of $F_{d_{j}}\left(x\right)$ is:

*H_j_* = −∑*P_ij_lnP_ij_* (5)

For every value *d_j_* in length data D, it has a corresponding entropy value *H_j_* in, which would achieve their maximum value at the optimal value *d_0_* in terms of the principle of maximum entropy. 

This article also compared the value of 3/$\sqrt{2}$*d_0_*, about which in [23] it is argued that the threshold value *d_0_* should multiply with 3/$\sqrt{2}$ based on the definition of Gaussian distribution and the 3-sigma rule [36].

### 2.3. Data

This paper takes Wuhan city, located in central China, as a case study area. As one of the nine National Center Cities of China, Wuhan is the most populous city in Central China. The city boasts abundant mountain and water resources and is divided into “Three Towns” known as Wuchang, Hankow, and Hanyang by the Yangtze River and Han river. The complicated geographic condition makes the identification of natural city boundaries more important rather administration boundaries. 

Three kinds of POI data collected as voluntary geographical information in Wuhan during 2016 was taken from the Baidu Map API, which provides open data of volunteer geographic information in the popular App Baidu Map in China. Such data included restaurants (*n =* 14,233), convenience stores (*n =* 9819) and banks (*n =* 4487). As a comparison, the Mobile Phone (MP) base stations (*n* = 33,758) were utilized as well. This data was provided by a partner telecommunication operator whose market share was about 60%, and verified to be proportionally representative of the whole population distribution in Wuhan [37].

## 3. Results and Discussion

### 3.1. Generation of the Service Area

**Figure 5:**
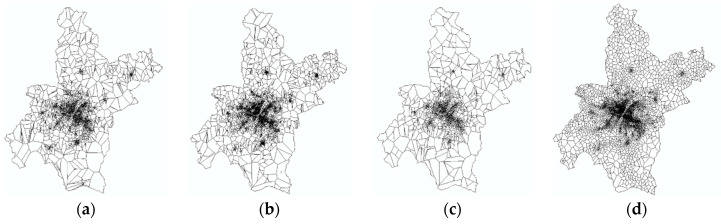
Urban expansion diagrams based on gridded population distribution. (**a**) Restaurants; (**b**) Stores; (**c**) Banks; (**d**) MP base stations.

The service area could be constructed in ArcGIS with the Thiessen polygon toolbox followed by a Clip operation of the administrative boundaries of Wuhan (Figure 5). All four service area maps show the feature of a fractal or heavy tail, wherein there are far more small polygons than large polygons [38]. Moreover, as the location varies from urban center to rural area, the polygons become larger. It is noteworthy that service area of mobile phone base stations shows a more even feature than those of POIs, where the former is planned by the government and the latter is built by the invisible hand of the market.

Statistical analysis was performed on the service areas of different POIs (Table 1). The amount of points in the generated polygons is less than the original ones, especially, the base station data lose the most, with a proportion of up to 2/3. This is mainly due to the fact that many base stations are located very close to each other [39], so most of them were ignored in the operation of generating Thiessen polygons. Based on Table 1, restaurant and mobile phone base stations possess the minimum service areas with 8 m^2^ and 12 m^2^ respectively, showing high density in the central city, meanwhile, the base station has a much smaller amount in maximum than POIs, further indicating its relative evenness in spatial distribution.

**Figure 6:**
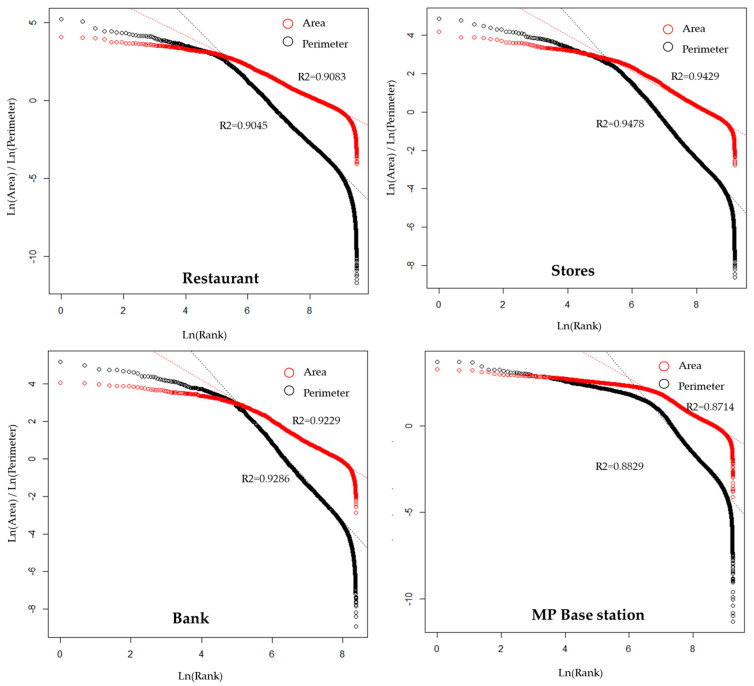
Logarithmic Rank-Size distribution of service area.

As the head/tail break method has indicated that the fractal features inside an urban complex system constitute a Zipf distribution in rank-size of the human physic environment, a logarithmic rank-size transformation would show a linear fit. Such an assumption was consistent with all the four datasets, wherein stores gives the highest R^2^, then banks and restaurants, and mobile phones (MP) base stations had the lowest (Figure 6).

### 3.2. Seeking the Threshold Value with the MaxEnt Method

**Figure 7:**
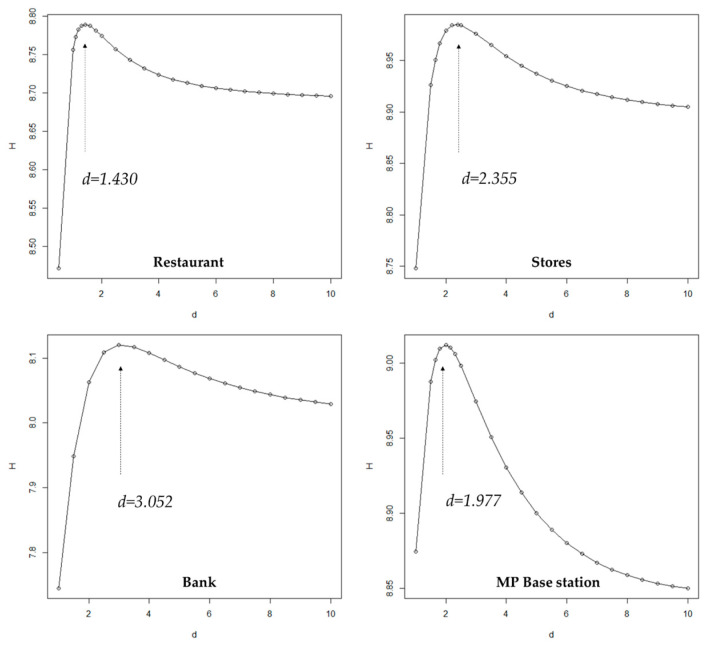
Maximum Entropy threshold value.

The next step is extracting such polygons to generate natural city boundary with an optimal threshold value *d_j_* that make the entropy of *F_d_(x)* achieve the maximum value of *H_j_* (Equation (5)). When *d_j_* varies from the minimum value to the maximum, the entropy value H of all above four kinds of data increased in the beginning, and then decreased slowly after reaching a peak (Figure 7). The value at peaks are the corresponding maximum entropy threshold values. Interestingly, the threshold values are close to the mean values, which are used in the head/tail breaks method (Table 2).

### 3.3. Comparison of Natural City Boundaries by Different Threshold Values

With such thresholds, three possible natural boundaries were formed, all the polygons were symbolized by the three break value of mean (head/tail breaks), MaxEnt threshold (H) and 3/$\sqrt{2}$H (3σ principle) (Figure 4, Table 3). For further exploration, the natural city boundary created using road junctions by Beijing City Lab (BCL) was added to test and compare the accuracy [40].

All different types of POIs show a much smaller boundary than the administrative boundaries of Wuhan City (Table 3), indicating the natural city boundaries of various human activities. Although the amount of restaurants (n = 14,233) is the largest, its smallest boundary implies that the restaurants are more likely located near places with a higher density of population. Oppositely, the stores located more evenly in the urban area generate the largest amount of natural city area. As for the banks which have been planned by the government or commercial institutions, they show a middle level between stores and restaurants. What type of POIs and which threshold length is utilized to extract service areas are crucial to defining the natural city boundary, identifying the real vitality of human activities.

**Figure 8:**
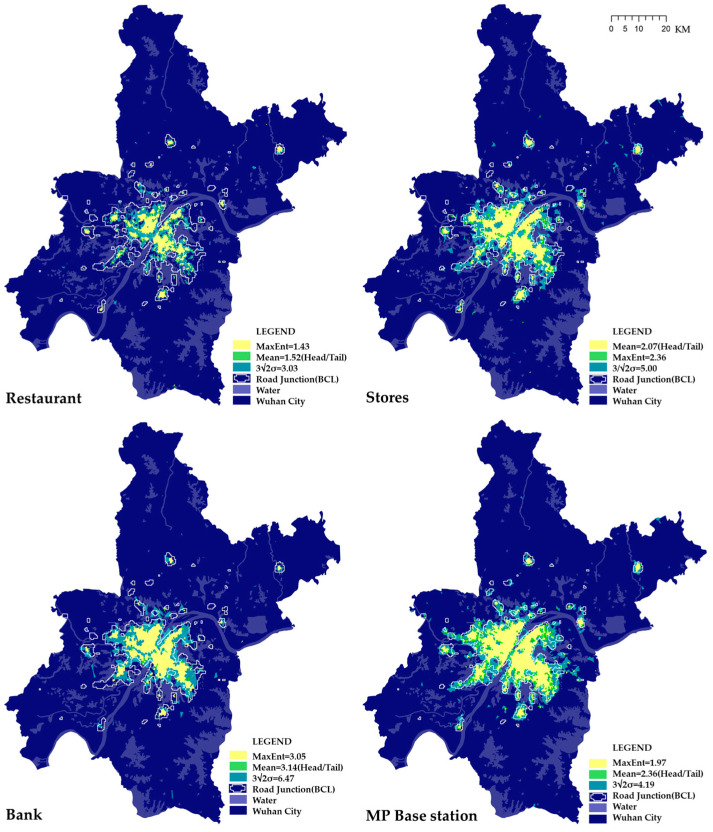
Natural city boundaries extracted by threshold values.

It is easy to find that the natural city boundaries delimitated by MaxEnt and Mean are very close, both of which are very different from the one generated by BCL road junctions, whereas the 3/$\sqrt{2}$ multiple values of MaxEnt appears approximately the same as that one (Figure 8). Such consistency demonstrates that the MaxEnt threshold value and 3σ principle are applicable to delimitate the natural city boundaries on a city level. It is noteworthy that the POI of stores shows the most similarity to the road junction data, rather than the mobile phone base stations. Such a result reveals the different feature between them: the stores emerged in a bottom-up way, while base stations were planned by the government. Moreover, although the number of restaurant is larger than that of stores, its boundary is smaller, indicating that the spatial distribution of stores is more suitable to reflect the density of human activities. It is also understandable that stores provide services all over the cities other than restaurants, most of which seldom located in industrial parks, for example.

**Figure 9:**
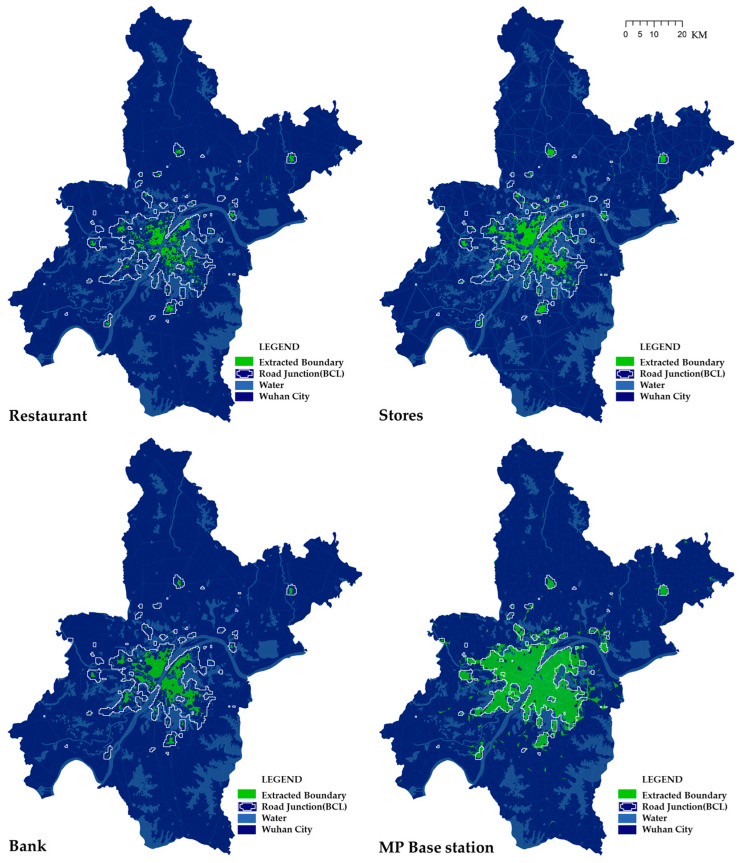
Natural city boundaries extracted by TIN and head/tail breaks.

The method utilized in [21] has also been implemented, wherein the boundaries are much smaller than the POI ones (Figure 9, Table 3). Furthermore, the boundary generated by MP base stations is close to the boundary of the road junctions, which verified the previous assumption that both MP base stations and street networks are constructed by the government in an up-down way, which may not match the reality of population density in the new city of East Wuhan.

As shown in Table 3, it is possible to delimitate natural city boundaries by extracting the service area of POIs with the maximum entropy threshold value and the principle of 3σ on a city scale, showing better suitability than the head/tail breaks method.

**Figure 10:**
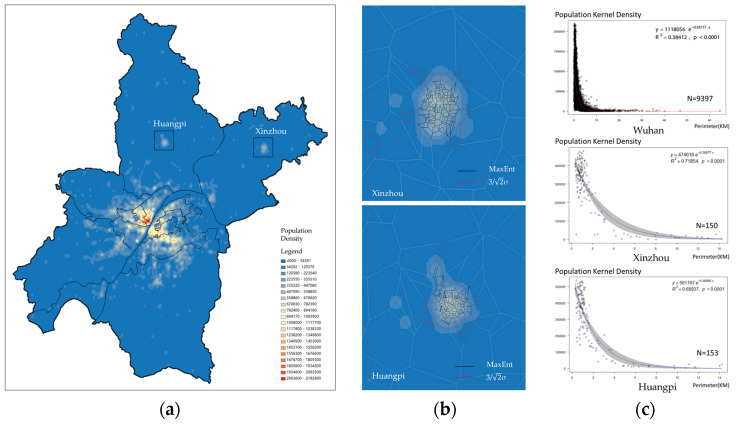
Relevance of population density and perimeters of service area. (**a**) Kernel density of mobile phone population; (**b**) Sample Subcenters; (**c**) Perimeter vs population of SA.

To further verify the correlation of population density and the distribution of POIs, the service area perimeters of POIs was compared with the population density generated by the user population of mobile phone stations based on the kernel density method (Figure 10a), which has been proved to have higher resolution than traditional census data [39]. The kernel density map shows a polycentric structure in Wuhan, wherein the density maps of the two relatively isolated subcenters, Huangpi and Xinzhou, are consistent with the service area of stores extracted by MaxEnt (Figure 10b). Such approximation indicates the feasibility of SA-MaxEnt. Meanwhile, the exponent function of the population density and perimeters of service areas show a high fitness with R^2^ = 0.72 in Xinzhou and 0.69 in Huangpi (Figure 10c). In other words, the higher the population density, the smaller the perimeters of the service area.

## 4. Discussion

Jiang utilized mixed POIs to generate basic urban geographic units with TINs, extracting urban area units based on head/tail breaks which took the mean as the threshold value, providing an innovative and convenient way for delimiting natural city boundaries with volunteer geographic information. Simultaneously, such a method also evokes three questions: are TINs is the only POI-based geographic units? Is the mean the best threshold value? What are the differences among natural city boundaries proposed by various kinds of POIs?

Because POIs provide services for the surrounding population within the city, this paper supposes utilizing service area as the basic natural city units based on Reilly’s Law of Retail Gravitation [19], although there exist differences in service capacities, the vast number of POIs make it possible to ignore the influence, which makes Thiessen polygons able to present its service area. According to the heavy tail and self-organized feature of such polygons in the context of urban complex systems [41], the principle of maximum entropy is implemented to seek the critical value which mostly influences the deviation of service areas. As the maximum entropy shows the same distribution as a normal distribution, the 3σ principle of Gaussian distribution is also tested to achieve an appropriate threshold value for extracting the service area polygons. Based on the comparison of the experimental result based on such two methods with BCL boundaries determined by road junctions, SA–MaxEnt method shows better accuracy than TIN-heat/tail breaks at the city level.

Different kinds of POIs also show various natural city boundary outputs, indicating their differences in service and surrounding population density. The POIs of stores show the best performance in delimiting the natural city boundary, showing that the distribution of stores is more matched with the density of human activities than restaurants and banks. In other words, the spatial distribution of retail stores is more related to the surrounding population density, due to which it may serve for much longer time. Moreover, the boundary of mobile phone base stations is larger than that of POIs, which reveals its up-down feature other than the bottom-up characteristics of POIs, although it has a more even spatial distribution and a larger number. Such a result also manifests that the accuracy of the natural city boundaries is up to the proper kind of POI rather than its amount. 

The natural city boundary given by stores POI appears to be similar to that generated by road junctions, and the delimitation is better than the result of head/tail breaks and TIN, which use the mean as the threshold value to aggregate TIN.

Utilizing the maximum entropy principle to seek a hidden value underlying the complexity of service areas could be seen as an exploratory application for entropy to solve the uncertainty in the mass complex system [31], transform the power law distribution into the normal distribution, displaying the inner fractal order in human activities in urban complex systems [35]. 

## 5. Conclusions

The main contribution of this paper is the SA-MaxEnt method which provides an innovative way to delimit natural cities with POIs, which could be applied to identify regional structures, hierarchy and urban sprawl, especially in terms of new cities or ghost cities, street networks, as remote sensing data and light maps may fail to reflect the reality of urban vitality. Secondly, this paper revealed that the geographic units generated by the Thiessen polygon method were better than by TIN, which indicates that the service-based POIs such as stores could present a more direct relation with the surrounding people served, showing a distribution of human activities density based on Reilly’s Law. Furthermore, the principle of maximum entropy was introduced to calculate the possible threshold value, showing its powerful potential in solving the uncertainty problem in the urban complex system. It is also noteworthy that the threshold value 3/$\sqrt{2}$*d_0_* gave a more similar boundary with road intersections rather than *d_0_*, which may need more exploration. 

Undoubtedly, the research does have limitations. The main weakness of the current study lies in that POIs still have limitations in representing human activities, such as in newly built industrial parks where there exist many workers but less service facilities. Furthermore, the whole approach was applied to a single city, and comparisons among cities could be conducted in future studies. Moreover, the use of the principle of maximum entropy in solving the uncertainty problem in urban contexts should be further explored.

## Figures and Tables

**Table 1:** Service area and perimeter statistics of POI and mobile phone stations.

	Restaurant	Stores	Bank	MP Base Station
Amount	13207	9678	4289	10212
Service Area	(km^2^)			
Min area	0.000008	0.000180	0.000133	0.000012
Max area	185.819	130.447822	180.126999	42.908001
Mean	0. 650851	0.888088	2.002382	0.842049
Standard Error	4.5672	14.107599	9.164582	2.361717
Perimeter	(km)			
Min Perimeter	0.017092	0. 061354	0. 057230	0.016712
Max Perimeter	57.826401	64.695572	59.245899	27.1527
Mean	1.520791	2.074611	3.13577	2.358477
Standard Error	3.530842	3.84682	5.797607	3.044902

**Table 2:** Threshold value comparison.

	Mean (M)	MaxEnt (H)	Ratio (H/M)	$3/\sqrt{2}H(3\sigma )$
Restaurants	1.520791	1.430	0.940300	3.0334876
Stores	2.074611	2.355	1.135152	4.9957086
Banks	3.13577	3.052	0.973285	6.4742686
MP Base Stations	2.358477	1.977	0.838252	4.1938496

**Table 3:** Area of Natural city boundaries delimitated by the different threshold values.

	Mean(M)	MaxEnt(H)	$3/\sqrt{2}H$	TIN-Head/Tail Breaks
Restaurants	204.62 km^2^	186.24 km^2^	427.18 km^2^	240.47 km^2^
Stores	337.76 km^2^	388.77 km^2^	815.77km^2^	134.44 km^2^
Banks	282.21 km^2^	271.33 km^2^	617.9 km^2^	199.10 km^2^
MP Base Stations	536.48 km^2^	424.07 km^2^	1017.832 km^2^	846.17 km^2^
Road Junctions (BCL)	807.34 km^2^			
